# Assessment of hematological parameters of petrol filling workers at petrol stations in Gondar town, Northwest Ethiopia: a comparative cross-sectional study

**DOI:** 10.1186/s12199-020-00886-1

**Published:** 2020-08-29

**Authors:** Sisay Getu, Elias Shiferaw, Mulugeta Melku

**Affiliations:** 1 Department of Medical Laboratory Sciences, College of Medicine and Health Sciences, Debre Tabor University, Debre Tabor, Ethiopia; 2 Department of Hematology and Immunohematology, School of Biomedical and Laboratory Sciences, College of Medicine and Health Sciences, University of Gondar, Gondar, Ethiopia

**Keywords:** Hematological parameters, Blood, Petrol, Ethiopia

## Abstract

**Background:**

Petrol is the non-specific term for petroleum which is used for inside combustion of engines. Petrol filling workers are highly vulnerable to occupational exposure to these harmful substances which lead to hemato-toxicity and blood disorders such as leukemia, aplastic anemia, and dysplastic bone marrow. Thus, this study was aimed to assess hematological parameters of petrol filling workers in Gondar town, Northwest Ethiopia.

**Methods:**

A comparative cross-sectional study was conducted from January to March 2019 in Gondar town, Northwest Ethiopia. A total of 110 study participants comprising 55 study groups and 55 controls group were recruited by a convenient sampling technique. Socio-demographic data were collected using a structured questionnaire, and 3 ml of venous blood was collected for the determination of hematological parameters. The data were entered into Epi info 7.2.0.1 and analyzed by SPSS version of 20. Mean, standard deviation, median, and interquartile ranges were used to present the data. Independent *t* test and Mann-Whitney *U* test were used to compare the mean or median difference between parametric and non-parametric hematological parameters, respectively. Moreover, Pearson product-moment and Spearman’s rank-order bivariable correlations analyses were used to describe the correlation between hematological parameters and duration of exposure to petrol. A *P* value of ≤ 0.05 was considered statistically significant.

**Results:**

The study revealed that mean red blood cell count and hemoglobin level as well as the median hematocrit, mean cell hemoglobin concentration, platelet count, absolute lymphocytes count, and red cell distribution width values of petrol filling workers showed a significant increment compared with the control group. On the other hand, the mean cell hemoglobin value of petrol filling workers showed a significant decrement compared with healthy controls. Moreover, the duration of exposure to petrol showed a significant positive correlation with red blood cell count and mean cell hemoglobin concentration; however, a significant negative correlation was observed with mean cell volume.

**Conclusion:**

This study showed that the majority of hematological parameters of petrol filling workers showed an increment compared with healthy controls which might be associated with exposure to petrol chemicals. However, further longitudinal study with a larger sample size should be conducted to explore the impact of petrol exposure on hematopoiesis.

## Introduction

Petrol includes crude oils and different refined oil products. It is a significant source of polycyclic aromatic hydrocarbons. After extraction, crude oil is transported to an oil refinery where complex hydrocarbon compounds are separated and converted through different refining operations for being useable fuel sources. Finally, impurities are eliminated through chemical treatment of each product [1]. Benzene is one of the natural constituent of crude oil as well as a product of petrol refining which is emitted in large quantities from oil refineries. Benzene is one of the main carcinogens among more than 165 occupational carcinogens which are identified by the International Agency for Research on Cancer (IARC). However, the pathogenic potential of petrol remains inconclusive and not yet fully understood [2, 3].

Although the role of benzene in the etiology of various hematopoietic malignancies requires accurate further explanation, the IARC report indicated the possible association of benzene with non-Hodgkin’s lymphoma, multiple myeloma, and other hematopoietic malignancies [4]. Gasoline is also one of the crude oil products which are well-thought-out as environmental pollutants and toxicants. It has adverse effects on organs like kidney, lung, and liver. Due to the heavy metals contained in gasoline, its vapor inhalation has been reported to change hematological parameters and induce anemia through bone marrow hypoplasia [5].

The particles generated from petrol exhaust are extremely small in diameter and large in surface area. They can carry a much larger fraction of toxic compounds, such as hydrocarbons and metals on their surface. These particles are deposited in large amount and deeper into the lung. As a result, the transport of oxygen to cells is slow down as they form methemoglobin, resulting in functional anemia [6]. Benzene metabolism is taken place in the liver and bone marrow; hence, these are the main sites for benzene toxicity. Benzene metabolite covalently binds to cellular macromolecules lastly results in dysfunction of bone marrow. Thus, long-term exposure to benzene leads to consistent structural and numerical chromosomal aberrations in lymphocytes and bone marrow cells [7, 8].

According to the study done in Iran, benzene and ethylene oxide are common carcinogens in the workplace. The estimated incidence of leukemia in the general population is reported to be 8.5 and 6.1 cases per 100,000 populations for males and females, respectively [9]. More than two million workers are exposed to benzene each year and result in malignant and non-malignant disorders worldwide [10].

The United States National Cancer Institute in collaborated with the Chinese Academy of Preventive Medicine studied that workers exposed to benzene developed lympho-hematopoietic malignancies in China [11]. In Great Britain from (1990–1993) years, 29,800 people were exposed to benzene carcinogen agent [12]. In New Zealand workplaces, benzene is one of 50 known human carcinogens [13].

Petrol filling workers are exposed to petrol carcinogenic agent through inhalation of vapors, contamination of particles during eating and drinking, and skin contact with particles from petrol vapors [14, 15]. Thus, occupational exposure leads to hemato-toxicity and blood disorders such as blood cancer (leukemia), aplastic anemia, and dysplastic bone marrow [16]. Besides, occupational exposure to petrol vapors has lethal effects on a variety of organs like heart, lung, skin, and kidney and systems such as respiratory, immune, and nervous systems which in turn causes various diseases [17].

A complete blood count (CBC) is a test that provides information about the distribution and quantity of blood cells. This test provides valuable information about the kinds and the numbers of cells in the blood particularly red blood cells (RBC), white blood cells (WBC), and platelets that indicates health professionals to check symptoms, like weakness, fatigue, or bruising. In addition, it also diagnoses pathologic conditions such as anemia. It can reflect acute or chronic infection allergies and problems with clotting. So, CBC is an easy and readily available screening tool for evaluating hemato-toxicity of benzene [18]. Thus, the current study was designed to investigate the effects of petrol exposure on hematological parameters of work in petrol filling.

## Materials and methods

### Study design, setting, and period

A comparative cross-sectional study was conducted among petrol filling workers at Gondar town petrol stations in comparison with apparently healthy blood donors at the University of Gondar comprehensive specialized referral hospital blood bank. Gondar is located in the northwestern part of Ethiopia, in the Amhara region state with 180 km far from Bahir Dar, the capital city of the regional and 727 km from Addis Ababa, the capital city of Ethiopia. Based on the 2007 national census conducted by the Central Statistical Agency of Ethiopia, Gondar town has an estimated total population of 206,987 people of whom 98,085 are males and 108,902 females [19]. There are 17 petrol stations with a total of 81 workers. Of thus workers, 52 are males and 29 are females. The study was done from January to March 2019.

### Sample size and sampling technique

According to the rule of thumb that has been recommended by van Voorhis and Morgan, 30 participants per group are mandatory to detect real differences between groups, which could lead to about 80% power [20]. Therefore, 110 study subjects which comprise 55 petrol filling workers as a case and 55 healthy blood donors as control were recruited into this study. Study participants in both cases and controls were taken from the study population by a convenient sampling technique.

### Source and study population

All petrol station workers who were exposed to petrol chemicals at Gondar town petrol stations and all apparently healthy blood donors at the University of Gondar comprehensive specialized referral hospital blood bank during the study period were as the source population for cases and controls, respectively. All petrol filling workers at Gondar town petrol stations and apparently healthy blood donors at the University of Gondar comprehensive referral hospital blood bank who fulfilled the inclusion criteria were included in the study.

### Inclusion and exclusion criteria

All petrol filling workers with age ranges of 18–65 years who were exposed to petrol fo a minimum of one year and all petrol non-exposed apparently healthy blood donors with age ranges of 18–65 years were included in the study. However, individuals who have ever had a history of either chewing chat or smoking cigarette; reactive for human immunodeficiency virus (HIV), hepatitis B virus, and hepatitis C virus; and individuals who had a history of inherited bleeding disorders, hypertension, chronic renal disease, chronic liver disease, and malignancy were excluded from the study. In addition to this, those who were on/taking cytotoxic chemotherapy, radiotherapy, and corticosteroid medications had also been excluded from the study. Moreover, individuals who were with evidence of ill health, in the use of medications, received a blood transfusion in the last 6 months, and donated blood within the last 3 months were excluded.

### Operational definitions

Definition and classification of hematological parameters were based on the local reference ranges [21]. The normal reference range for different hematological parameters was defined as follows: RBC 3.53–6.93 × 10^12^/l for male and 3.45–6.25 × 10^12^/l for female; hematocrit (Hct) 36.2–58.6% for male and 32.1–56.6% for female; hemoglobin (Hb) 11.5–18 g/dl for male and 11.0–16.0 g/dl for female; mean cell hemoglobin concentration (MCHC) 29.5–34.4% for male 28.5–34.4% for female; and mean cell hemoglobin (MCH) 26.6–33.3 pg for male and 25.8–32.8 pg for female, respectively. On the other hand, parameters such as WBC, absolute neutrophils, absolute lymphocytes, absolute mixed cell, platelet, mean cell volume (MCV), red cell distribution width (RDW), neutrophil %, lymphocyte %, and mixed cell % were defined as 3.2–8.8 × 10^9^/l, 1.6–5.1 × 10^9^/l, 1–3.5 × 10^9^/l, 0.2–1 × 10^9^/l, 128–432 10^9^/l, 85–100 fl; 12–17%, 36–69%, 22–55%, and 0.2–1%, respectively for both adult males and females [21]. Hematological parameters below or above these pre-stated reference ranges were considered as decreased or increased in accordance.

### Data Collection and laboratory methods

#### Socio-demographic data

Socio-demographic data of the study participants were collected via face-to-face interview using a pre-tested structured questionnaire. The questionnaire consists of variables regarding socio-demographic characteristics of the study participants and duration of exposure to petrol chemicals for petrol filling station workers.

#### Clinical data

Clinical information of the study participants was collected using a structured data checklist. Physical diagnosis and detailed history had been taken by experienced nurses to assess a history of inherited bleeding disorders, hypertension, chronic renal disease, chronic liver disease, malignancy, medication, cigarette smoking, chat chewing, blood transfusion, and blood donation.

#### Laboratory analysis

A total of 3 ml of venous blood was collected using a sterile needle and syringe to determine hematological parameters. Hematological parameters were analyzed by Sysmex KX-21 (Sysmex Corporation, Kobe, Japan), three differential automated hematology analyzer. In addition, all study participants were screened for HIV, hepatitis B virus, and hepatitis C virus. All laboratory procedures were conducted according to standard operating procedures.

### Data quality assurance

The questionnaire was prepared in English, translated to Amharic language, and then translated back to English to check for consistency. The training was given to data collectors prior to data collection. The data were collected by trained data collectors: experienced nurses and medical laboratory technologists. The process of data collection was closely supervised by investigators.

### Data analysis and interpretations

After data was cleaned and checked for completeness, it was entered in Epi info version of 7.2.0.1 and transferred to SPSS 20.0 for analysis. Normality was checked via visual plots like the Q-Q plot, the statistical method like the Kolmogorov-Smirnov normality test, and the Shapiro-Wilk normality test. Homogeneity of variance was checked by the Levene test. For normally distributed data, the independent *t* test was used to compare differences in the mean of hematological parameters between cases and controls. For non-normally distributed data, the Mann-Whitney *U* test was used. To measure the correlation of hematological parameters with duration of exposure to petrol, the Pearson product-moment bivariate correlation for normally distributed data and Spearman’s rank-order correlation analysis for non-normally distributed data were used. The results were summarized in mean ± standard deviation (SD), median, and interquartile range (IQR). The results were presented in tables and texts. *P* value ≤ 0.05 was considered as statistically significant for all analysis.

### Ethical considerations

The study was conducted after ethical approval was obtained from the Research and Ethical Review Committee of School of Biomedical and Laboratory Sciences, College of Medicine and Health Sciences, the University of Gondar. Support and permission letters were obtained from the Gondar town health office and from each petrol station administrative offices. Moreover, written informed consent was taken from study participants after explaining the purpose, benefits, and possible risks of this study. Participation in the study was purely on a voluntary basis. Study participants who had abnormal hematological results were linked to the University of Gondar comprehensive specialized referral hospital for better medical advice and healthcare services.

## Results

### Socio-demographic characteristics of study participants

A total of 110 age- and sex-matched study participants were included in this study. The median (IQR) age was 28 (22–35) years and 29 (22-34) years for cases and controls, respectively. However, there were no statistically significant differences between case and control subjects in age (*P* = 0.983). Among 55 cases, the majority 41 (74.5%) were males and 28 (50.9%) were single in their marital status (Table 1).

**Table 1:** Socio-demographic characteristics of study participants

Variables	Socio-demographic characteristics	Study group (*n* = 55)		Control group (*n* = 55)	
		Frequency	Percentage (%)	Frequency	Percentage (%)
Sex	Male	41	74.5	41	74.5
	Female	14	25.5	14	25.5
Age group (years)	20–30	34	61.8	34	61.8
	31–40	14	25.5	14	25.5
	41–50	5	9.1	5	9.1
	51–60	2	3.6	2	3.6
Address	Gondar	52	94..5	Journal 49	89.1
	Around Gondar	3	5.5	6	10.9
Marital status	Single	28	50.9	32	58.2
	Married	22	40	23	41.8
	Widowed	5	9.1	0	0
Religion	Orthodox	45	81.8	46	83.6
	Muslim	9	16.4	1	1.8
	Protestant	1	1.8	8	14.8
Educational status	Unable to read and write	2	3.6	0	0
	Primary school	8	14.5	0	0
	Secondary school	29	52.7	0	0
	Above secondary school	16	29.1	55	100
Petrol exposure in (year)	1–5	31	56.3	-	-
	6–10	4	7.3	-	-
	> 10	20	36.4	-	-

### Hematological parameters of study participants

The magnitude of leukocytosis, leukopenia, and neutropenia among petrol filling workers was 14.5%, 3.6%, and 18.2%, respectively. On the other hand, anemia and neutropenia among healthy controls were reported to be 3.6% and 7.3%, respectively (Table 2).

**Table 2:** Hematological parameters of study participants

		Study group (*N* = 55)		Control group (*N* = 55)	
Hematological parameters	Category	Frequency	Percentage (%)	Frequency	Percentage (%)
WBC (× 10^9^/l)	Below normal	2	3.6	0	0
	Normal	45	81.8	55	100
	Above normal	8	14.5	0	0
RBC (× 10^12^/l)	Below normal	0	0	0	0
	Normal	55	100	55	100
	Above normal	0	0	0	0
Hb (g/dl)	Below normal	1	1.8	2	3.6
	Normal	52	94.6	52	94.6
	Above normal	2	3.6	1	1.8
Hct (%)	Below normal	1	1.8	3	5.4
	Normal	54	98.2	52	94.6
	Above normal	0	0	0	0
MCV (fl)	Below normal	26	47.3	6	10.9
	Normal	29	52.7	49	89.1
	Above normal	0	0	0	0
MCH (pg)	Below normal	4	7.3	0	0
	Normal	51	92.7	55	100
	Above normal	0	0	0	0
MCHC (%)	Below normal	0	0	0	0
	Normal	34	61.8	53	96.4
	Above normal	21	38.3	2	3.6
Platelet (× 10^9^/l)	Below normal	0	0	0	0
	Normal	55	100	100	100
	Above normal	0	0	0	0
Neutrophil (%)	Below normal	9	16.4	1	1.8
	Normal	44	80	50	90.9
	Above normal	2	3.6	4	7.3
Neutrophils absolute (× 10^9^/l)	Below normal	10	18.2	4	7.3
	Normal	38	69.1	43	78.2
	Above normal	7	12.7	8	14.5
Lymphocyte (%)	Below normal	0	0	2	3.6
	Normal	51	92.7	53	96.4
	Above normal	4	7.3	0	0
Lymphocytes absolute (× 10^9^/l)	Below normal	0	0	0	0
	Normal	54	98.2	55	100
	Above normal	1	1.8	0	0
Mixed cells (%)	Below normal	5	9.1	5	9.1
	Normal	31	56.4	40	72.7
	Above normal	19	34.5	10	18.2
Mixed absolute (× 10^9^/l)	Below normal	6	10.9	1	1.8
	Normal	39	70.9	50	90.9
	Above normal	10	18.2	4	7.3
RDW (%)	Below normal	2	3.6	4	7.3
	Normal	52	94.5	51	92.7
	Above normal	1	1.8	0	0

### Comparison of hematological profiles of petrol filling workers and the control group

The mean RBC count and Hb level were significantly higher in petrol filling workers as compared with the control group. On the other hand, the mean MCV value of petrol filling workers has shown a significant decrement compared with healthy controls. Moreover, median HCT, MCHC, platelet count, absolute lymphocytes count, and RDW of petrol filling workers were higher than health controls (Table 3).

**Table 3:** Comparison of hematological parameters of study participants

Hematological parameters	Study group (cases) (*n* = 55)	Control group (*n* = 55)	*P* value
RBC (× 10^12^/l)^☼☼^	5.38 ± 0.57	4.75 ± 0.52	< 0.001
Hb (g/dl)^☼☼^	15.51 ± 1.61	13.77 ± 1.54	< 0.001
MCV (fl)^☼☼^	85.12 ± 3.79	88.77 ± 3.62	< 0.001
WBCs (× 10^9^/l)^☼^	6.10 (2.4)	6.00 (3.2)	0.995
Hct (%)^☼^	46.6 (4.5)	42.2 (6.4)	< 0.001
MCH (Pg)^☼^	29.3 (1.5)	29.2 (1.8)	0.530
MCHC (%)^☼^	34.2 (1.3)	32.7 (1.1)	< 0.001
Platelet (× 10^9^/l)^☼^	258 (98)	222 (81)	0.005
Neutrophil (%)^☼^	52.0 (18.8)	53.2 (10.5)	0.068
Neutrophils absolute (× 10^9^/l)^☼^	3.00 (2)	3.1 (2.05)	0.357
Lymphocyte (%)^☼^	37.9 (17.5)	35.3 (11.6)	0.188
Lymphocytes absolute (× 10^9^/l)^☼^	2.20 (0.9)	2.00 (0.6)	0.037
Mixed (%)^☼^	9.30 (7.9)	9.30 (4.5)	0.253
Mixed absolute (× 10^9^/l)^☼^	0.6 (0.4)	0.6 (0.2)	0.538
RDW (%)^☼^	13.7 (0.9)	13.1 (1.1)	< 0.001

☼☼Data were expressed as mean (± SD)

^**☼**^Data were expressed as median (IQR)

### Correlation analysis

Based on the distribution of the hematological parameters, Pearson product-moment and Spearman’s rank-order bivariable correlation analyses were done for normally distributed and non-normally data, respectively (see Additional File 1). Duration of petrol exposure showed a significant positive correlation with RBC count and MCHC value. On the other hand, duration of exposure for petrol had shown a significant negative correlation with MCV result of petrol filling workers. However, other hematological parameters indicated non-significant positive correlations (Table 4). The additional files showed the scatter plot of the bivariate correlation analysis of RBC count, MCV, and MCHC with duration of exposure for petrol (see Additional File 2, Additional File 3, and Additional File 4).

**Table 4:** Correlations of duration of petrol exposure with hematological parameters of petrol station workers

Parameters	Correlation coefficient [*p*(*r*), (rho)]	*P* value
RBC (× 10^12^/l)	0.287*	0.034
MCV (fl)	− 0.321*	0.017
MCHC (%)	0.337**	0.012

*Pearson product moment correlation *p*(*r*)

**Spearman’s rank-order correlation coefficient (rho)

## Discussion

Health effects of occupational exposure to petrol chemicals are fairly unfamiliar among petrol filling workers. However, continued exposures for petrol chemicals might be the cause of well-known consequence of bone marrow suppression, hematological lethal effect, and cancer. Petrol containing solvents like benzene cause hemato-toxicity, immune-toxicity, neurotoxicity, and carcinogenicity [22]. Thus, the current study was designed with the aim to assess hematological parameters of petrol filling workers at Gondar town petrol stations.

According to this study, mean RBC and Hb values of petrol filling workers showed a significant increment compared with healthy controls. A consistent result had been reported from India [23], Iran [24], Iraq [25], China [26], Palestine [27], and Bulgaria [28]. In addition, our result showed a weak positive correlation between RBC count of petrol filling workers with duration of exposure which is in agreement with the previous study from China [26]. On the other hand, our result was contrary to studies from India [27] and Egypt [29]. Variation in the sample size, study period, socio-demographic variables, and duration of exposure might be the possible reason for the discrepancies.

The current study also showed a significant elevation in the median value of HCT among the exposed group compared with the control group. Our finding was in agreement with earlier studies conducted in India [23] and Nigeria [1]. However, studies carried out in Bulgaria [28] and Egypt [29] were not agreed with our finding. In these two previous studies, years of exposure and smoking habits of the study subjects were not considered. This might be the probable reason for variations.

In general, the possible reason for an increment in RBC parameters of petrol filling workers might be due to the fact that petrol is known to contain high concentrations of carbon monoxide which can enter the blood via the respiratory system. The molecule had a high affinity for Hb up to 200 times compared with oxygen to form carboxyhemoglobin which interferes with blood’s oxygen transport capacity and results in tissue hypoxia. Tissue hypoxia stimulates erythropoiesis which finally leads to the production of more number of RBC, in turn, rises of Hb and Hct levels [30]. Moreover, metabolisms of benzene yield quinone metabolite, which able to give rise to reactive oxygen species formation. A rise to reactive oxygen species leads to an abnormal condition like hypoxia. Hypoxia stimulates the production of more erythropoietin by the kidney and liver. These situations indeed lead to the formation of more number of RBC, a rise of Hb and Hct level in the peripheral blood [31].

Our result revealed that the mean MCV value of petrol filling workers showed a significant decrement compared with the control group. A consistent result had been reported from Sudan [18], Korea [32], and Nigeria [33, 34]. Evidence suggested that a reduction in the cellular size could be due to the membrane alterations. Benzene may have an impact on flexibility and permeability of the cell although its mechanisms are not clearly known [35]. Moreover, as a result of the metabolism of petrol chemicals leads to the formation of free radicals, it damages the cell membrane [35].

Our study also revealed that the median platelet count of petrol filling workers showed a significant increment compared with healthy controls which was supported by a study from UK [36]. This might be due to petrol chemicals may catalyze the activity of the bone marrow for more platelet production [37]. On the other hand, our result was contrary to previous studies from India [23] and France [38]. Thus, contradicted findings might be due to other confounding factor like HIV and hepatitis virus infections which were not excluded in previous studies.

According to our result, the median of the MCHC level of petrol filling workers showed a significant increment compared with healthy controls. The result was in agreement with a study from Gaza [39]. However, our result was contrary to findings from UK [36] and Iran [40] that reported a lower MCHC value in the exposed group than the control group. The possible reason for this discrepancy might be due to variation in duration of exposure. In this study, a majority of study participants had an exposure history of < 5 years compared with a study from Iran [40] which includes participants with a minimum exposure history of 5 years in which duration of exposure had an impact. On the other hand, the nature of the study design, cross-sectional versus retrospective, might be the other possible cause for the discrepancy. Evidence showed that an increase in MCHC can be a valuable clinical indicator of increased spherocytosis which is noticed upon exposure to benzene [35].

Our study also showed that the median absolute lymphocyte count of petrol filling workers was higher than health controls which was supported by a study from Nigeria [1]. However, our result was in contradiction with previous studies from India [8, 27]. Variation in sample size may be the possible reason for this discrepancy. In our study, a majority of cases are exposed for < 5 years; early exposure petrol oil may elicit the immune activation and production of lymphocyte. Along with such exposure, they may be more susceptible to infectious diseases like infectious mononucleosis that increase the lymphocyte population [1].

Moreover, according to our result, the median RDW value of petrol filling workers showed a significant increment compared with healthy controls. RDW is a key indicator which principally indicates impaired erythropoiesis and abnormal red blood cell destruction. However, it is also associated with inflammation and allergic reaction which might be due to benzene. RDW is a strong predictor of all-cause, cardiovascular and cancer-related mortality [41].

## Limitation and strength of the study

Since the study was conducted among small sample size, this might reduce the statistical power of the study. On the other hand, the cross-sectional nature of the study design makes it difficult to establish a cause-effect relationship. Given these limitations, the physical diagnosis was carried out to assess vital signs and other comorbidities which might have an impact on hematological parameters. In addition, all study participants were screened for HIV, hepatitis B virus, and hepatitis C virus.

## Conclusions

Findings in this study lead to conclude that the majority of hematological parameters (RBC, Hb, Hct, MCHC, RDW, absolute lymphocyte count, and platelet count) of petrol filling workers showed an increment compared with healthy controls which might be associated with the effect of petrol. On the other hand, the MCV value of petrol filling workers indicated a decrement compared with healthy controls. Moreover, duration of petrol exposure showed a significant positive correlation with RBC and MCHC values while a negative correlation with that of MCV level of petrol filling workers which can suggest the effect of this substance on hematological parameters. Therefore, it is important to assess change in hematological parameters by further longitudinal study with larger sample size using an inclusive manner of advanced tests (molecular, cytogenetic, immuno-histochemistry, and biochemistry) to rule out the cause-effect relationships between petrol exposures and change in hematological parameters which may help to prevent associated hematological disorders and complications thereof.

## Supplementary information

**Additional file 1.** Shows the output of RBC, MCV and MCHC data normality statistics, which includes Kolmogorov-Smirnov and Shapiro-Wilk tests.

**Additional file 2.** Shows the scatter plot of the bivariate correlation analysis of RBC count with duration of exposure for petrol.

**Additional file 3.** Shows the scatter plot of the bivariate correlation analysis of MCV with duration of exposure for petrol.

**Additional file 4.** Shows the scatter plot of the bivariate correlation analysis of MCHC (%) with duration of exposure for petrol.

## Data Availability

All data and materials were included in the manuscript

## Abbreviations

BMI: Body mass index
CBC: Complete blood count
EDTA: Ethylenediaminetetraacetic acid
ESR: Erythrocyte sedimentation rate
Hct: Hematocrit
Hb: Hemoglobin
IARC: International Agency for Research on Cancer
IQR: Interquartile range
MCHC: Mean cell hemoglobin concentration
MCH: Mean cell hemoglobin
MCV: Mean cell volume
RBCs: Red blood cells
RDW: Red cell distribution width
SD: Standard deviation
WBCs: White blood cells
