# Supplementary material for: Assessment of hematological parameters of petrol filling workers at petrol stations in Gondar town, Northwest Ethiopia: a comparative cross-sectional study
Source: Environ Health Prev Med. 2020 Aug 29;25:44. doi: 10.1186/s12199-020-00886-1 (PMC7456503; doi:10.1186/s12199-020-00886-1)
Supplement: Supplementary file 1 — Additional file 1. Shows the output of RBC, MCV and MCHC data normality statistics, which includes Kolmogorov-Smirnov and Shapiro-Wilk tests. [file 12199_2020_886_MOESM1_ESM.docx]

**Additional file 1:** Test of normality for RBC, MCV and MCHC values in case and controls

| **Tests of Normality** | | | | | | | | | | | | | |
| --- | --- | --- | --- | --- | --- | --- | --- | --- | --- | --- | --- | --- | --- |
|  | Kolmogorov-Smirnov^a^ | | | | | | Shapiro-Wilk | | | | | |  |
|  | Statistic | | df | | Sig. | | Statistic | | Df | | Sig. | |  |
| Case RBCs x 10^12^/l | .097 | | 55 | | .200^*^ | | .981 | | 55 | | .542 | |  |
| Case MCV(fl) | .077 | | 55 | | .200^*^ | | .966 | | 55 | | .122 | |  |
| Case MCHC% | .174 | | 55 | | .000 | | .729 | | 55 | | .000 | |  |
| *. This is a lower bound of the true significance. | | | | | | | | | | | | |  |
| 1. Lilliefors Significance Correction | | | | | | | | | | | | |  |
| **Tests of Normality** | | | | | | | | | | | | | |
|  | | Kolmogorov-Smirnov^a^ | | | | | | Shapiro-Wilk | | | | | |
|  |  | Statistic | | Df | | Sig. | | Statistic | | df | | Sig. | |
| Control RBCs x10^12^/l | | .059 | | 55 | | .200^*^ | | .988 | | 55 | | .841 | |
| Control MCV(fl) | | .079 | | 55 | | .200^*^ | | .971 | | 55 | | .205 | |
| Control MCHC% | | .066 | | 55 | | .200^*^ | | .968 | | 55 | | .148 | |
| *. This is a lower bound of the true significance. | | | | | | | | | | | | | |
| a. Lilliefors Significance Correction | | | | | | | | | | | | | |
