# Supplementary material for: Assessment of hematological parameters of petrol filling workers at petrol stations in Gondar town, Northwest Ethiopia: a comparative cross-sectional study
Source: Environ Health Prev Med. 2020 Aug 29;25:44. doi: 10.1186/s12199-020-00886-1 (PMC7456503; doi:10.1186/s12199-020-00886-1)
Supplement: Supplementary file 2 — Additional file 2. Shows the scatter plot of the bivariate correlation analysis of RBC count with duration of exposure for petrol. [file 12199_2020_886_MOESM2_ESM.docx]

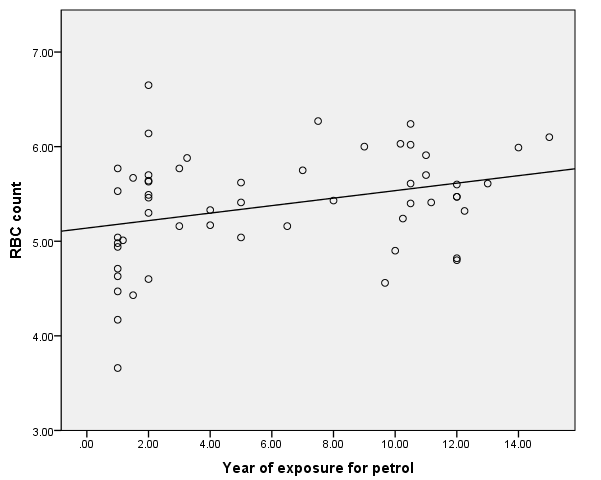


**Additional File 2:** Pearson product moment bivariate correlation plot of RBC(x10^12^/L) and duration of exposure for petrol among patrol station workers
